# Waste Valorization via *Hermetia Illucens* to Produce Protein-Rich Biomass for Feed: Insight into the Critical Nutrient Taurine

**DOI:** 10.3390/ani10091710

**Published:** 2020-09-21

**Authors:** Alessia Giannetto, Sabrina Oliva, Kristian Riolo, Domenico Savastano, Vincenzo Parrino, Tiziana Cappello, Maria Maisano, Salvatore Fasulo, Angela Mauceri

**Affiliations:** 1 Department of Chemical, Biological, Pharmaceutical and Environmental Sciences, University of Messina, Viale F. Stagno d’Alcontres 31, 98166 Messina, Italy; soliva@unime.it (S.O.); kristianriolo92@hotmail.com (K.R.); vparrino@unime.it (V.P.); tcappello@unime.it (T.C.); mmaisano@unime.it (M.M.); sfasulo@unime.it (S.F.); amauceri@unime.it (A.M.); 2 Progetto Hermetia, 89015 Reggio Calabria, Italy; domenicosavastano@hotmail.it

**Keywords:** food waste bioconversion, BSF, protein, amino acids, gene characterization, gene expression

## Abstract

**Simple Summary:**

The increasing demand of nutrients for food and feed imposes the urgent need to implement current nutritional resources while finding valuable alternative sources of fats and proteins. The present study aims to evaluate the efficiency to bioconvert the substrate proteins of vegetable wastes into valuable larval biomass by the insect Black Soldier Fly (BSF), *Hermetia illucens*. Here, we report that BSF larvae and prepupae show a high protein content characterized by different profiles of valuable amino acids, including taurine, a crucial nutrient for animal feed and future fish aquaculture. Moreover, we provide insights into the genetic basis of taurine biosynthesis in BSF for the first time and we show that the regulation of the genes associated with taurine synthesis influences the taurine content in BSF larvae and prepupae. These findings on peculiar BSF phenotypes encourage the utilization of larvae and/or prepupae to meet different nutritional requirements of fish species as alternative source of relevant amino acids including taurine. Notably, the bioconversion process by BSF represents a sustainable and economically interesting joint solution to meet the protein demand for animal and aquafeed in the next decades as well as a sustainable biotechnological tool for vegetable waste valorization.

**Abstract:**

Insects have been recognized as sustainable alternative sources of nutrients for food and feed. The Black Soldier Fly (BSF), *Hermetia illucens*, is a particularly promising species for its great potential in the waste valorization to produce, during the bioconversion process, high-value fat and proteins that currently represent a valuable source for fish feed. The present study aims to evaluate the efficiency to use substrate proteins in two different BSF developmental stages as sustainable biotechnological tools for vegetable waste management. We provide insights into the nutritional values of both V instar larvae and prepupae in terms of valuable amino acids with special focus on taurine, a crucial nutrient for fish. Moreover, we cloned four key genes from BSF involved in the taurine biosynthesis pathway, *2-aminoethanethiol dioxygenase* (*Hiado*), *cysteine dioxygenase* (*Hicdo*), *cysteine sulfonate decarboxylase* (*Hicsad*), and *glutamate decarboxylase* (*Higad*). The gene expression analysis in larvae and prepupae by qPCR showed development-specific profiles suggesting they influence the taurine content during BSF development. These findings showed peculiar phenotypes in larvae and prepupae that can be selected for different biotechnological applications as sustainable source of relevant amino acids and taurine to support the increasing demand for animal feed and aquafeed in the next decades.

## 1. Introduction

Worldwide, insects are recognized as potential solutions to face the increasing demand for alternative resources of nutrients, mainly proteins and fat, in the future.

Insect biomass can provide more than 30% crude protein on a dry matter basis and is commonly characterized by amino acid profiles that can meet the nutritional requirements of livestock: the high content of lysine, threonine, and methionine offers a valuable alternative source compared to the soybean meal that, at the present, is the main protein-rich source for livestock nutrition [1,2]. The inclusion of insects in feed formulation has been generally demonstrated not unfavorably affecting growth performance nor quality in a wide range of animal species [3].

Although legislative aspects concerning the use of insect proteins to feed aquaculture animals (Regulation EU No. 2017/893 amending Regulations EC 999/2001 and EU No. 142/2011) have been encouraging the inclusion of insect-based diets, in Western countries concerns on the safety of insects as feed and food are still subsisting. The Black Soldier Fly (BSF), *Hermetia illucens* (Diptera: Stratiomydae), is one of the species with the highest potential for mass production and represents a sustainable and economically interesting solution to meet the protein demand and to manage wastes, simultaneously. Literature from the last decade has demonstrated that BSF during the larval development constitutes a concrete and valuable source of high-quality protein that can be incorporated in animal feed [4,5] for livestock diets [3,6,7] and economically relevant fish species [1,8,9]. Most studies on the use of BSF-based fish diets have shown that BSF during the larval stages provides a suitable protein resource for animal feed since health and growth performance are not usually affected by the inclusion of BSF meal [1,10,11,12,13].

Notably, BSF can meet the need to replace fishmeal in aquafeeds as a potential source of taurine, a critical nutrient for fish aquaculture, whose supplementation in the diet has positive effects for several species on growth and digestive functions [14].

Taurine (2-aminoethanesulfonic acid), an amino sulfonic acid which is not incorporated into proteins and constitutes the free amino acid pool, is generally considered as a semi-essential amino acid in most animal species. In fish, it is involved in vital physiological processes including osmoregulation, modulation of neurotransmitters, anti-oxidant function, and vision, and it has been shown to stimulate growth in several species, such as *Oncorhynchus mykiss* [15], *Paralichthys olivaceus* [16], *Psetta maxima* [17], *Pagrus major* [18], and *Salmo salar* [19].

In vertebrate, the major biosynthetic pathway of taurine (2-amino ethanesulfonic acid) starts from the two sulphur-containing amino acids methionine and cysteine, whose oxidation to cysteine sulfinic acid by cysteine dioxygenase (CDO) is followed by decarboxilation to hypotaurine by cysteine sulfonate decarboxylase (CSAD) and subsequent oxidation to taurine. Additionally, hypotaurine can be produced by the oxidation of cysteamine by 2-aminoethanethioldioxygenase (ADO) and by the decarboxylation of cysteine sulfinic acid by glutamate decarboxylase (GAD) that also catalyzes the decarboxylation of cysteic acid to taurine [20]. Although taurine content has been rarely evaluated in insects [21,22], knowledge of the physiological mechanisms determining its amount could provide useful information to better exploit this crucial nutrient in animal feed. Moreover, molecular mechanisms underlying the taurine biosynthetic pathway in insects are far to be elucidated and it cannot be excluded that different taurine contents could reflect differences in the expression levels and/or activities of the key enzymes involved in its biosynthesis. Due to the urgent need to identify sustainable additive sources of proteins and amino acids to use in animal feed, it is imperative to evaluate the nutritional content of insects as well as assess the modification of most nutrient content according to the insect diet. Particularly, it has been shown that the nutritional composition of BSF strongly depends on the composition of the feeding media [1,3,23], but a comprehensive understanding of the effect of the larval diet on the nutrient content has not been achieved yet. Notably, recent studies reported that important changes in the proximate composition throughout the larval development may occur [5,24,25]. Liu et al. explored the fluctuations in the nutrient content during the whole life cycle showing marked variations in the nutritional composition depending on the developmental stage of BSF reared on chicken feed and found a reduction of protein content with age together with an increasing of dry matter content in the later instars [24]. More recently, focusing on the late larval developmental stages able to provide an high yield in terms of biomass, Giannetto et al. evaluated the BSF V instar larvae and prepupae nutritional composition demonstrating important differences in the proximate composition with special concern on fatty acid profiles; the fascinating opportunity to exploit these peculiarities ad hoc was suggested to meet specific industrial requirements [26]. Although, these late developmental stages have been evaluated for their nutritional composition and for their potential use in animal feed [8,27], the distinctive traits of V instar larvae and prepupae still need to be better clarified in order to take advantage of their potential peculiar properties in animal feed as well as to formulate species-specific diets in aquaculture.

In this study, we evaluated the efficiency to bioconvert vegetable waste proteins into valuable biomass by BSF as a sustainable biotechnological tool for waste valorization. Particularly, BSF larvae and prepupae were assessed for their potential as eco-friendly and economically advantageous sources of alternative proteins and important amino acids as well as taurine to use as feed. In addition, taurine biosynthetic pathway was investigated to better understand the molecular basis of taurine content that can be exploited to match the nutritional requirements of livestock and fish diets in aquaculture.

## 2. Materials and Methods

### 2.1. H. Illucens Culture and Experimental Design

The stock colony of *Hermetia illucens* (www.progettohermetia.it—Italy) was reared at 27–32 °C and >70% relative humidity as previously described [26]. Hatched larvae were reared on a mix of fruit and vegetable wastes (40% pears, 45% banana, 5% tomatoes, 10% various leafy green vegetables) after grinding. BSF V instar larvae and prepupae were recognized following the published criteria [28]. In particular, the observation of exuviae of each instar larvae was used to determine the different 6 instars; the body weight, the head capsule width, and the cuticle color were evaluated for recognizing the selected larval stages. V instar samples were collected from three replicate tanks (body weight 70.2 ± 16.5 mg FW; head capsule width 0.92 ± 0.02 mm) or were allowed to grow until they had changed (in color) from cream to black/brown prepupae [29] in other three replicate tanks (body weight 180.2 ± 23.6 mg FW; head capsule width 1.15 ± 0.08 mm). Three biologically independent samples were collected from each replicate of the two analyzed developmental stages. After harvesting, the V instar larvae and prepupae samples were weighed and stored for further analyses.

### 2.2. Nutritional Composition and Protein Yield

The nutritional values, namely, dry matter (DM), crude protein (CP), crude fat (CF), acid detergent fibers (ADF), neutral detergent fibers (NDF), and ash, were obtained from three replicates for each sample using the AOAC techniques (2005); the crude protein values were corrected for chitin content according to [30] as extensively reported by [26]. The efficiency of conversion of digested feed (ECD_F_) was calculated as [final larvae dry weight – initial larval dry weight] (g)/[total dry feed offered – dry residue remained] (g). In order to investigate the protein yield at the end of the bioconversion process, the protein conversion ratio (PCR = FCR X% feed protein, divided by 100) and the protein efficiency ratio (PER = FCR X% feed protein, divided by % BSF protein, where FCR = feed added (g) divided by total biomass (g)) were calculated on a dry matter basis for larvae and prepupae [31].

### 2.3. Determination of Amino Acid Profile

Pooled samples of dried and ground V instar larvae, prepupae, and rearing substrate (40 grams from each pool) were subjected to amino acid analysis by an automated amino acid analyzer (HPLC) (Beckman system gold HPLC 126AA, USA) after hydrolyzing the sample with 6 N HCl for 22 h at 110 °C [32] via an analytical service laboratory ISO 9001:2015 certified.

### 2.4. Minerals and Heavy Metals Analyses

Minerals were analyzed following the method described by AOAC [33]. The samples were dried and ground. The powder samples were digested with 1:3 HNO_3_:HCl at 200 °C for 30 min. After filtration, minerals were determined in each sample through an inductively coupled plasma-mass spectrometry (ICP-MS, Agilent, USA) via an analytical service laboratory ISO 9001:2015 certified.

### 2.5. Identification of the Genes Associated with Taurine Biosynthesis

To investigate the taurine metabolic pathway in *Hermetia illucens*, the genome database Kyoto Encyclopedia of Genes and Genomes (KEGG) (http://www.genome.jp/kegg/pathway.html) was used. The genes associated with the taurine and hypotaurine metabolism pathway in *Musca domestica* and *Drosophila melanogaster* were used for BLAST search against the NCBI database (https://blast.ncbi.nlm.nih.gov). Retrieved sequences coding for *cysteine dioxygenase* (*cdo*), *cysteine sulfonate decarboxylase* (*csad*), *2-aminoethanethiol dioxygenase* (*ado*), and *glutamate decarboxylase* (*gad*) from Diptera species (Table S1) were used to design degenerate primers (Table S2) for cloning of the respective homologues from *H. illucens.* Total RNA was extracted with TRIzol Reagent (Invitrogen) and treated to remove genomic DNA contamination as detailed by [34]. One microgram of total RNA samples was reverse transcribed with the QuantiTect Reverse Transcription Kit (Qiagen). cDNAs were amplified by PCR using recombinant Taq DNA polymerase (Invitrogen) and the obtained PCR products were gel-purified by the E.Z.N.A Gel Extraction kit (Omega Bio-tek) for subsequent cloning and sequencing as detailed by [35]. BLAST search was performed to confirm the identities of the putative genes involved in taurine biosynthesis.

### 2.6. Sequence Characterization and Phylogenetic Analysis

Sequences of 2-aminoethanethiol dioxygenase (ado), cysteine dioxygenase (cdo), cysteine sulfonate decarboxylase (csad), and glutamate decarboxylase (gad) from *H. illucens* were submitted to GenBank. The deduced amino acid sequences were identified using the ExPASy Translate tool (https://web.expasy.org/translate) and ORFfinder (https://www.ncbi.nlm.nih.gov/orffinder). For each retrieved sequence, the protein functional domains were predicted by Conserved Domain Search (https://www.ncbi.nlm.nih.gov/Structure/cdd/wrpsb.cgi). Sequences were aligned using the multiple sequence alignments program Clustal W. Phylogenetic analyses of the identified key genes associated with taurine biosynthesis and their orthologs from NCBI reference sequences (Table S1) were conducted in MEGA X [36] using the Neighbor-Joining method with the bootstrap test (1000 replicates) and Dayhoff correction model.

### 2.7. mRNA Levels of the Taurine Biosynthesis Genes

Quantitative PCR (qPCR) was used to evaluate the mRNA levels of the putative genes associated with the taurine biosynthesis in BSF larvae and prepupae. Transcript levels of *ado*, *cdo*, *csad*, and *gad* were quantified using the QuantiTect SYBR^®^Green PCR Kit (Qiagen), 1:20 diluted cDNA samples, and gene-specific qPCR primers (Table S2) with a Rotor-Gene Q 2 plex Hrm thermocycler (Qiagen). The amplification efficiency and the specificity of each reaction were evaluated as reported elsewhere [37]. For each reaction, six biological replicates were run in duplicate together with minus reverse transcriptase and no template controls. Raw data of target genes were corrected using the normalization factor calculated by the GeNorm Software from three suitable reference genes, *elongation factor* (*ef1-α*), *18s ribosomal RNA* (*18s rRNA*), and *16s ribosomal RNA* (*16s rRNA*), as described by [38].

### 2.8. Statistical Analysis

The data were subjected to analysis of variance followed by Student–Newman–Keuls post hoc tests by using the SPSS software 16.0 (SPSS Inc.) in order to assess statistically significant differences between BSF V instar larvae and prepupae. A *p*-value of less than 0.05 was considered as statistically significant.

## 3. Results

### 3.1. Nutrient Content of BSF V Instar Larvae and Prepupae and Efficiency of Protein Use

**Figure 1:**
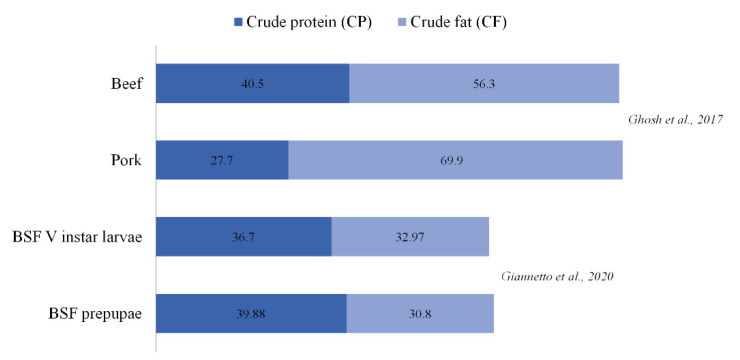
Comparison of protein and fat content (% dry matter, DM basis) of Black Soldier Fly (BSF) V instar larvae and prepupae with published data (Giannetto et al., 2020; Ghosh et al., 2017).

**Figure 2:**
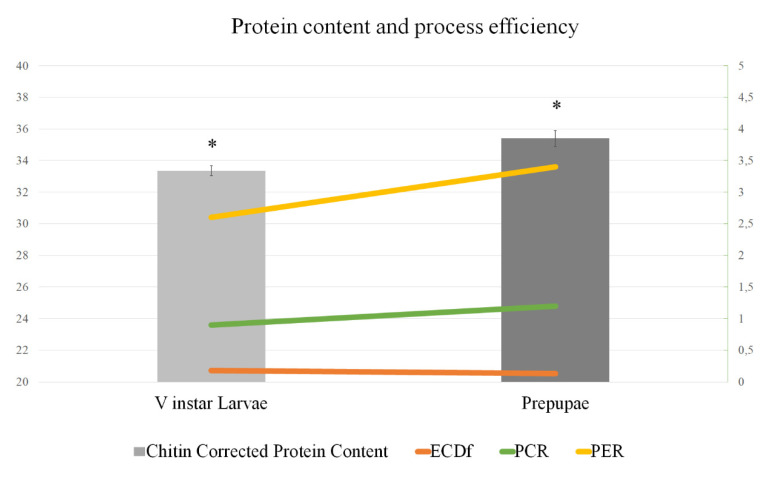
Protein content and efficiency of protein use. Chitin-corrected protein content (% dry matter, DM basis), efficiency of conversion of digested feed (ECD_F_), protein conversion ratio (PCR), and protein efficiency ratio (PER) of BSF larvae and prepupae. Asterisk (*) indicates statistically significant differences in chitin-corrected protein content between V instar larvae and prepupae (*p* < 0.05).

To evaluate the nutritional potential of the two developmental stages, BSF V instar larvae and prepupae were assessed for their proximate composition. Interestingly, protein and fat contents were comparable to conventional food sources of animal origin (Figure 1). The protein content of V instar larvae (36.70%) was slightly lower than of prepupae (39.88%). The same protein content trend between V instar larvae and prepupae was still observed when analyzing chitin-corrected crude protein values (33.35% vs. 35.40%, respectively) (Figure 2). The calculated efficiency of conversion of digested feed (ECD_F_) was 0.18 in V instar larvae and 0.13 in prepupae showing that earlier developmental stages present a higher efficiency of conversion and consequently better performance in converting the metabolized rearing substrate into valuable biomass. Conversely, the protein conversion ratio (PCR) values were higher in prepupae in respect to larvae (1.2 vs. 0.9, respectively); a similar trend was observed for the protein efficiency ratio (PER) that was 3.4 and 2.6 in prepupae and larvae, respectively (Figure 2).

### 3.2. Amino Acid Profile of BSF V Instar Larvae and Prepupae

The amino acid composition of BSF V instar larvae and prepupae was determined to further asses the nutritional value of the two developmental stages in the perspective of using *H. illucens* as animal feed. The analysis of profiles showed there were some differences in the amino acid composition during the BSF larval development (Table 1), but most of the essential amino acids for fish were found in *H. illucens* regardless of the developmental stage. The content of essential amino acids was comparable between BSF V instar larvae and prepupae (29.34% vs. 30.52%), except for leucine and valine that were more abundant in V instar larvae than prepupae (2.25% vs. 1.79% and 3.04% vs. 2.59%, respectively) while the amount of arginine and histidine was higher in the prepupae (6.49% vs. 8.87% and 7.49% vs. 8.12%). Different values were observed in the content of the non-essential amino acids whose total value was higher in V instar larvae than prepupae (46.92% vs. 35.95%, respectively). Among the non-essential amino acids, glutamic acid was the most abundant one in both the developmental stages. Remarkably, the amino sulfonic acid taurine, contained in the free amino acid pool, was found in both developmental stages although at different levels with prepupae containing the highest concentration (45 mg/Kg vs. 24 mg/Kg in V instar larvae).

### 3.3. Minerals and Heavy Metals of BSF V Instar Larvae and Prepupae

The levels of minerals such as calcium, phosphorus, potassium, magnesium, iron, zinc, and manganese together with the amount of other toxic and heavy metals (arsenic, cadmium, mercury, lead) were measured in BSF V instar larvae and prepupae in order to assess their mineral composition and the potential bioaccumulation during *H. illucens* larval development (Table 2). The mineral profiles of the two evaluated developmental stages were comparable with higher levels of all the minerals, except the heavy metals, in prepupae than in V instar larvae. Indeed, the amount of these detrimental elements, such as arsenic, cadmium, mercury, and lead, decreased during BSF larval development, with concentrations within the admitted limit.

### 3.4. Molecular Characterization of the Genes Associated with Taurine Biosynthesis

The reference pathway of taurine and hypotaurine metabolism from *Musca domestica* was used to identify the genes associated with taurine metabolism in *Hermetia illucens*. The four key genes herein identified were designated as *Hiado*, *Hicdo*, *Hicsad*, and *Higad* and submitted to GenBank (Accession numbers: **MT180129**, **MT180130**, **MT180131**, and **MT180132**, respectively).

**Figure 3:**
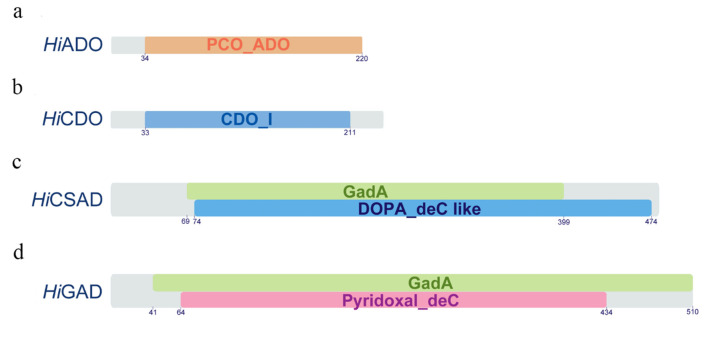
Schematic representation of *Hermetia* *illucens* proteins involved in taurine biosynthesis. Conserved domains of proteins characterized in this study: (**a**) *Hi*ADO: PCO_ADO domain (orange); (**b**) *Hi*CDO: CDO_I domain (blue); (**c**) *Hi*CSAD: GadA (green) and DOPA_deC like (blue) domains; (**d**) *Hi*GAD: GadA (green) and Pyridoxal_deC (purple) domains.

The *Hiado* cDNA consisted of 660 bp nucleotides coding a putative protein of 220 amino acids and characterized by the conserved functional PCO_ADO superfamily (accession cl06746) with the PCO_ADO domain (accession pfam07847) in the amino acid interval 34–220 (Figure 3; Figure S1). The respective deduced amino acid sequence of 2-aminoethanethiol dioxygenase from *H. illucens* showed high identity with those of other Diptera species, such as *Bactrocera dorsalis* (48.2%), *Ceratitis capitata* (46.2%), *Musca domestica* (45%), and also with ADO proteins from *Homo sapiens* (33.8%) and *Danio rerio* (34.6%).

The *Hicdo* cDNA consisted of 705 bp nucleotides coding a putative protein of 235 amino acids and characterized by the conserved functional cupin-like superfamily (accession cl21464) with the CDO_I domain (accession pfam05995) in the amino acid interval 33–211 (Figure 3; Figure S2). The respective deduced amino acid sequence of cysteine dioxygenase from *H. illucens* showed high identity with those of other Diptera species, such as *Rhagoletis zephyria* (59.7%), *Stomoxys calcitrans* (58%), *Musca domestica*, and *Drosophila serrata* (57.2%), and also with CDO proteins from *Homo sapiens* (48.7%) and *Danio rerio* (51.2%).

The *Hicsad* cDNA consisted of 1434 bp nucleotides coding a putative protein of 478 amino acids and characterized by the conserved functional Beta_elim_lyase superfamily domain (accession cl18945) in the amino acid interval 46-402 containing the DOPA_deC_like (accession cd06450, interval 74–474) and GadA (accession COG0076, interval 69–399) domains (Figure 3; Figure S3). The respective deduced amino acid sequence of cysteine sulfonate decarboxylase from *H. illucens* showed high identity with those of other Diptera species, such as *Ceratitis capitata* (60.9%), *Rhagoletis zephyria* (60.3%), *Stomoxys calcitrans*, and *Musca domestica* (60.1%), and also with CSAD proteins from *Homo sapiens* (54.5%) and *Danio rerio* (51.8%).

The *Higad* cDNA consisted of 1530 bp nucleotides coding a putative protein of 510 amino acids and characterized by the conserved functional Pyridoxal_deC domain (accession pfam00282) in the amino acid interval 64–434 contained within the specific GadA (accession COG0076) domain in the amino acid interval 41–510 (Figure 3; Figure S4).

The respective deduced amino acid sequence of glutamate decarboxylase from *H. illucens* showed high identity with those of other Diptera species, such as *Lucilia cuprina* (85.9%), *Musca domestica* (85.7%), *Drosophila melanogaster* (84.7%), and also with GAD proteins from *Homo sapiens* (58.2%) and *Danio rerio* (55.6%). The sequence homology was particularly high within the identified functional motifs.

**Figure 4:**
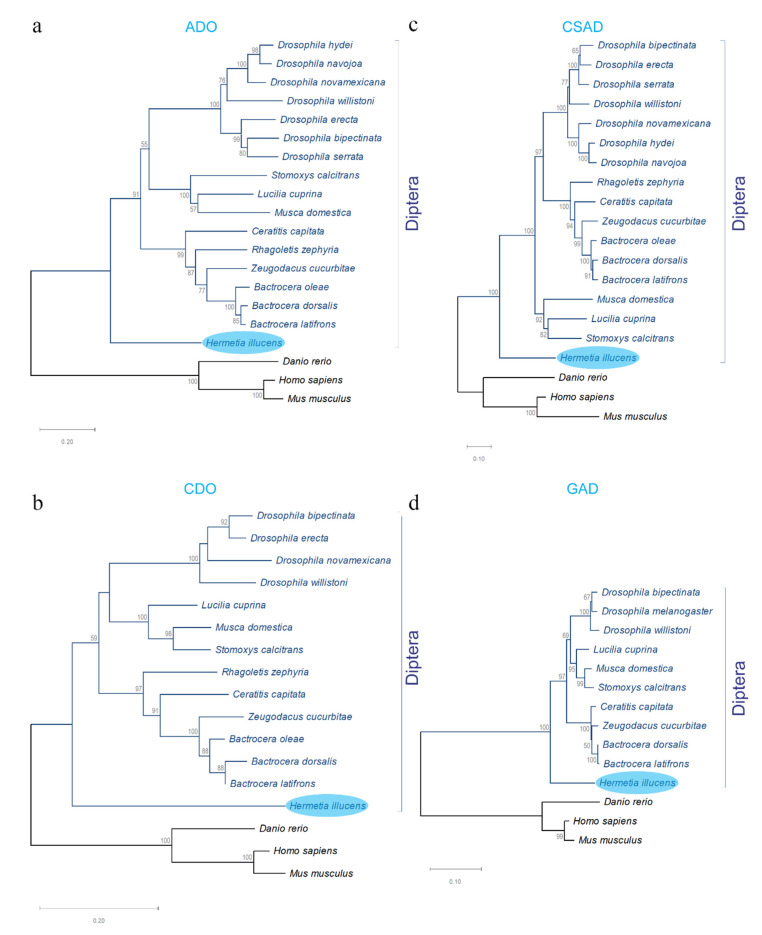
Phylogenetic analyses of *H. illucens* proteins with the relative orthologs from different species. Phylogenetic trees of (**a**) 2-aminoethanethioldioxygenase (ADO), (**b**) cysteine dioxygenase (CDO), (**c**) cysteine sulfonate decarboxylase (CSAD), and (**d**) glutamate decarboxylase (GAD) based on amino acid sequences were constructed using the Neighbor-Joining method. Bootstrap values (1000 replicates) are shown at each tree node. GenBank accession numbers of the protein sequences used in these analyses are reported in Table S1.

Phylogenetic trees of the four genes associated with taurine biosynthesis from *H. illucens* were constructed using the deduced amino acid sequences. The evolutionary relationships of *Hi*ADO, *Hi*CDO, *Hi*CSAD, and *Hi*GAD putative proteins with orthologs of other Diptera species and selected vertebrates (Table S1) showed that BSF proteins are closely related to homologous proteins from flies available in the NCBI database (Figure 4).

### 3.5. Quantitative PCR

**Figure 5:**
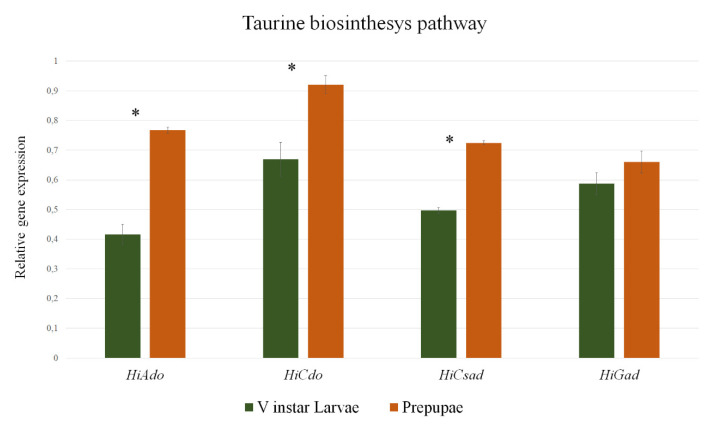
mRNA expression levels of the genes associated with taurine biosynthesis in *H. illucens*. Relative gene expression of *Hiado*, *Hicdo*, *Hicsad*, and *Higad* were evaluated by qPCR in BSF V instar larvae and prepupae. Results are presented as mean ± SD (*n* = 6). Asterisks (*) denote significant differences between the selected developmental stages (*p* < 0.05).

The mRNA levels of *H. illucens* *2-aminoethanethiol dioxygenase* (*Hiado*), *cysteine dioxygenase* (*Hicdo*), *cysteine sulfonate decarboxylase* (*Hicsad*), and *glutamate decarboxylase* (*Higad*) were quantified by real-time qPCR in BSF V instar larvae and prepupae (Figure 5). All the genes were expressed in both developmental stages although at different levels. In particular, *Hiado* mRNA levels were significantly higher in prepupae (two-fold) compared to V instar larvae (*p* < 0.01). The same pattern of expression was observed for *Hicdo*, *Hicsad*, and *Higad* showing increased transcript levels in the later developmental stage (1.4-fold, 1.5-fold, and 1.1-fold, respectively). The relative mRNA levels of *Hiado*, *Hicdo*, and *Hicsad* were significantly different between the two investigated developmental stages (*p* < 0.01), but no significant differences in *Higad* gene expression levels were observed between the BSF V instar larvae and prepupae, although *Higad* expression levels were slightly higher in prepupae.

## 4. Discussion

Insects have been recognized as a sustainable source of nutrients, mainly fat and proteins, for food and animal feeding [5,27,32,43,44,45,46,47,48]. Particularly, the Black Soldier Fly *Hermetia illucens* has been proposed in a list of insect species with the greatest potential as food and feed ingredients in Europe [49] and represents one of the most promising insect species as a valuable bioconverter of a broad range of organic substrates, including vegetable waste [1,47,50,51,52]. Recently, it has been receiving increased attention because of the high value nutrients obtained via this insect having been successfully used to produce aquaculture and livestock feeds. Indeed, this bioconversion process produces fat and high-value proteins that currently are being considered as a novel and renewable source of proteins for animal feed, and particularly for fish feed, while reducing waste biomass with consequent economic and environmental advantages [53,54]. Currently, the high quantity and quality of proteins required in terrestrial animal and fish diets are provided by fishmeal and soy meal but the environmental and economic repercussions of their use have been posing the urgent need to find alternative protein sources.

Studies on the nutritional value of *H. illucens* showed that its chemical composition varies depending on the rearing substrate [1,47,49,50,51,55,56] and the stage of development [24,26,45] with promising amounts of proteins, fatty acids, and macronutrients encouraging its use as animal feed [1,3,4,5,6,8,9,10,11,12,13,47] and for biodiesel production [57,58,59,60]. The protein content is one of the most important criteria to consider for feed protein sources, and the crude protein values recorded in the present study are comparable with those documented elsewhere for BSF [61,62]. It is worth mentioning that the reported protein concentrations of *Hermetia illucens* regardless of the developmental stage were similar to that of soybean meal [61] and within the protein requirements of most fish species that range from 25% (in freshwater species) to 55% (in marine species) [8]. These features make BSF an advantageous protein source in the animal feed industry, especially because the digestibility of insect-derived proteins is higher than that of vegetable proteins [61]. Remarkably, the differences in CP levels between V instar larvae and prepupae became significant when the crude protein values were corrected subtracting the chitin content, a nitrogen-containing polysaccharide in the exoskeleton of insects that deserves particular attention not only regarding the possible over-estimation of the protein content [30] but also as a component of digestibility to evaluate in the perspective of using BSF as a source of proteins in fish diets [8]. Although it has been documented that the chitin content could not be a possible hurdle of using insects in fish feeds [8], it is notable that the high levels of lauric acid contained in larvae and prepupae [26], due to its documented anti-inflammatory effects on the intestine [13], could mitigate the possible adverse effects of chitin in BSF-based fish diets. Nevertheless, the chitin that can be extracted from both larvae and prepupae constitutes a high-value biopolymer for the use in industrial applications, thus making *H. illucens* a promising alternative source of valuable biomass for obtaining relevant biomolecules next to lipids and proteins. Therefore, the sustainable bioconversion process by BSF larvae is particularly fascinating for waste valorization of low-value rearing substrates as vegetable waste. Notably, at the end of the bioconversion process, the values of both protein conversion ratio and protein efficiency ratio could reveal a higher protein yield in BSF at prepupal stage in respect to larvae. However, although the trend of the capacity to use substrate protein seems to increase from larvae to prepupae, a high difference is evident in PER values only. It can be inferred that the ratio of feed protein conversion to BSF biomass yield is not influenced by the developmental stage, but, on the other hand, the efficiency of conversion of substrate protein in BSF protein is higher when the prepupal stage is reached.

Since the quality of proteins to use as feed depends mainly on the amino acid composition, the nutritional value of BSF proteins was further evaluated by analyzing the amino acid profile of V instar larvae and prepupae, showing that both developmental stages are rich in relevant amino acids. In insects, the amino acid patterns are taxon-dependent, with Diptera showing amino acid compositions comparable with fishmeal that, due to the high protein content (up to 73%) and quality of essential amino acids, is the most important protein source in aquatic feeds [32]. The detection of dominant levels of glutamic acid in BSF is consistent with the findings reported elsewhere [24,30,39,63]. Although the amino acid requirements of each animal species are considerably different, the amino acids essential for fish are present in *H. illucens* V instar larvae and prepupae.

The content of essential amino acids in BSF was comparable with the levels measured in five commercial edible insects [41] as well as with the minimum requirement of amino acid levels in fish diets [40]. However, the content of some relevant amino acids for aquafeed was lower than the recommended levels for fish; these data are in agreement with the successful partial replacement of fish meal with BSF meal tested in different experimental diets of several commercial fish species [8,64,65].

To date, published reports on BSF-based diets brought evidences about the suitability of BSF during the larval stages as a valuable source for sustainable production of high-quality protein for fish species [8,9,10,11,12,13] as well as for animal feed [4,5] and for livestock diets [3,6].

Although amino acid levels were comparable in the two investigated developmental stages, interestingly, BSF prepupae biomass was characterized by a greater content of total free amino acids as well as higher taurine amounts.

Taurine (2-aminoethanesulfonic acid) has been reported as one of the most abundant free amino acids in the insect central nervous system [66]. Early studies on the taurine content in insects showed detectable amounts in tissues of the locust *Schistocerca americana gregaria* [67] and crickets [21]. However, the paucity of data available to date strongly suggests that taurine levels in insects are both species- and developmental stage-specific [21,22]. In the perspective of using BSF as feed, we investigated the levels of this critical nutrient for future fish and animal feeds. Interestingly, we reported significant levels of taurine in both V instar larvae and prepupae, with the later developmental stage showing the highest content.

To further investigate the physiological mechanisms determining these two different phenotypes, we here identified and characterized four key genes associated with taurine biosynthesis, namely, *2-aminoethanethiol dioxygenase* (*ado*), *cysteine dioxygenase* (*cdo*), *cysteine sulfonate decarboxylase* (*csad*), and *glutamate decarboxylase* (*gad*). These selected genes that are known to be involved in taurine biosynthesis in insects from the KEGG pathway database were characterized for the first time in *Hermetia illucens* and designated as *Hiado*, *Hicdo*, *Hicsad*, and *Higad*, respectively. Their sequences and phylogenetic analyses showed significant similarity of both cDNAs and deduced proteins with their relative homologues from Diptera species. Moreover, each of the identified amino acid sequences were found to contain specific conserved protein functional domain(s) that characterize homologous proteins from distant species including *Homo sapiens* and *Danio rerio*, strongly suggesting that the proteins associated with taurine biosynthesis and here identified play conserved functions during evolution. Phylogenetic analysis showed that the four identified proteins clustered with sequences of other Diptera and separately from selected mammals and fish.

The gene expression analysis of *Hiado*, *Hicdo*, *Hicsad*, and *Higad* showing different mRNA levels in V instar larvae and prepupae further supported their actual involvement in taurine biosynthesis during BSF larval development. The finding of detectable levels of taurine in both *H. illucens* V instar larvae and prepupae showed that Black Soldier Flies are able to synthesize this crucial amino acid and that its levels are modulated during development, as previously demonstrated for other insect species [21,22]. Moreover, the identification of the genes associated with taurine biosynthesis anticipate fascinating challenges to increase the taurine levels by modulating these genes in the future to meet the aquafeed requirements.

Beside the nutritional composition, the mineral content of BSF is an essential information in the perspective of using BSF as feeds. The mineral profile of *H. illucens* has been evaluated in a few studies [1,3,50] but early evidences suggest that the composition of the rearing substrate can influence the chemical composition of this insect species also in terms of mineral content [50]. According to previous studies reporting that the ash levels, closely related to the content of inorganic matter, are influenced by the developmental stage in *H. illucens*, we found that prepupae were characterized by a higher ash content than V instar larvae [26]. Among the minerals, calcium levels are particularly interesting as they are generally low in insects [68]. The obtained values from larvae and prepupae were comparable to those previously found in BSF [3,50]. The increase in Ca^2+^ from the larval to the prepupal stage could probably be imputable to the characteristic of BSF-mineralized exoskeleton that can incorporate calcium, as well as other minerals, into the cuticle [30]. These data firmly suggest that the mineral content cannot be exclusively influenced by the rearing substrate but also by the BSF developmental stage. The measured mineral profiles of BSF larvae and prepupae showed elevated levels of calcium, potassium, and phosphorus followed by a decreasing content of Mg > Mn > Fe > Zn that is in agreement with previous reports [50,62]. Particular concerns rise from the possible contamination with undesirable substances in *H. illucens* as animal feed; cadmium, lead, mercury, and arsenic in animal feed need to be monitored [69] to ensure animal and human health. Notably, the levels of toxic metals such as cadmium, arsenic, mercury, and lead were below the threshold level allowed in animal feed, as previously reported for cadmium and lead [70], and within the acceptable concentration in foods regulated by EU directive 2002/32/EC [42]. These results of mineral profiles give important information on the content of inorganic matters in BSF larvae and prepupae, thus suggesting the sustainability of using both developmental stages in the production of animal feed.

## 5. Conclusions

The present study provides additional information on the nutritional composition of *H. illucens* reared on vegetable waste contributing significantly to highlight the great potential of BSF as a valuable source of critical nutrients, including proteins and minerals that result from the bioconversion process of low-quality waste by BSF during larval development. Overall, we showed that the different phenotypes of BSF V instar larvae and prepupae arise from specific differences in protein metabolism and mineral content herein documented as well as the peculiar fatty acid profiles reported in our previous paper. The nutritional peculiarities of BSF V instar larvae and prepupae support the notable potential of both developmental stages as quality feed of commercial interest suggesting the intriguing chance to select favorable nutritional profiles to meet different industrial purposes.

Together, these findings strongly encourage the utilization of larvae and/or prepupae to meet different nutritional requirements of animal and fish species, as alternative sources of valuable amino acids including taurine, a critical ingredient in feed formulation.

## Figures and Tables

**Table 1:** Amino acid profile of BSF V instar larvae and prepupae. The essential amino acids for larvae are in italic [39]. ^h^ indicates essential amino acid for humans, ^f^ indicates essential amino acid for fish.

Amino Acid (%)	V Instar Larvae	Prepupae	Edible Insects *	Recommended Minimum Amino Acid Levels in Fish Diets ^#^
**Essential**				
*Arginine* ^f^	6.49 ± 0.2 ^a^	8.87 ± 0.5 ^b^	2.09–3.71	4.2–4.3
*Histidine* ^hf^	7.49 ± 0.4 ^a^	8.12 ± 0.7 ^b^	1.8–2.8	1.5–2.1
*Isoleucine* ^hf^	1.71 ± 0.02 ^a^	1.37 ± 0.07 ^a^	1.62–2.16	2.5–3.1
*Leucine* ^hf^	2.25 ± 0.1 ^a^	1.79 ± 0.02 ^b^	2.3–3.97	3.3–3.4
Lysine ^hf^	3.65 ± 0.2 ^a^	3.35 ± 0.2 ^a^	1.7–2.6	5.1–5.7
*Methionine* ^hf^	0.21 ± 0.04 ^a^	0.19 ± 0.1 ^a^	0–0.3	
Methionine and cystine				2.3–3.2
*Phenylalanine* ^hf^	1.28 ± 0.06 ^a^	1.03 ± 0.09 ^a^	1.62–1.83	
Phenylalanine and tyrosine				5–6.5
*Threonine* ^hf^	2.49 ± 0.3 ^a^	2.29 ± 0.07 ^a^	1.5–2	2–3.9
Tyrosine ^h^	0.73 ± 0.06 ^a^	0.92 ± 0.5 ^a^	2.6–3.7	
*Valine* ^hf^	3.04 ± 0.2 ^a^	2.59 ± 0.06 ^b^	2.7–3.2	2.8–3.6
**Non-essential**				
Alanine	16.49 ± 0.02 ^a^	11.67 ± 0.3 ^b^		
Aspartic acid	4.08 ± 0.4 ^a^	2.72 ± 0.08 ^b^		
Glutamic acid	21.76 ± 0.09 ^a^	17.74 ± 0.05 ^b^		
Glycine	2.08 ± 0.3 ^a^	2.02 ± 0.2 ^a^		
Serine	2.51 ± 0.07 ^a^	1.80 ± 0.06 ^b^		
Others	23.94 ± 0.1 ^a^	32.95 ± 0.05 ^b^		
**EAA**	**29.34** ± 0.2 ^a^	**30.52** ± 0.02 ^b^		
**NEAA**	**46.92** ± 0.3 ^a^	**35.95** ± 0.06 ^b^		
**Taurine (mg/Kg)**	**24** ± 0.02 ^a^	**45** ± 0.01 ^b^		

EAA, essential amino acid; NEAA, non-essential amino acid; Others, sum of proline, ornitine, asparagine, glutamine, γ-aminobutyric acid. ^#^ [40]; * [41]. Values are means ± SD. Different superscript letters (a and b) within the same row indicate significant differences for that given amino acid between larvae and prepuae (*p* < 0.05).

**Table 2:** Mineral and heavy metal contents of BSF V instar larvae and prepupae (Ca, P, K, Mg: % dry matter; Fe, Zn, Mn, As, Cd, Hg, Pb: mg kg^−1^, dry matter). The maximum content of undesirable substances allowed for animal feed use is reported [42].

Mineral	V Instar Larvae	Prepupae	EU Directive 2002/32/EC (mg/kg)
Ca	0.72	0.92	-
P	0.58	0.71	-
K	0.79	0.84	-
Mg	0.26	0.33	-
Fe	84	89	-
Zn	68	98	-
Mn	212	268	-
As	1.01	0.96	2
Cd	0.37	0.24	2
Hg	<0.05	<0.05	0.1
Pb	0.39	0.32	10

## Supplementary Materials

The following are available online at https://www.mdpi.com/2076-2615/10/9/1710/s1. Figure S1: *H. illucens* 2-aminoethanethiol dioxygenase (*Hi*ADO) protein sequence. Multiple sequence alignment of 2-aminoethanethiol dioxygenases from Diptera. The conserved residues in the amino acid sequences are highlighted in black; identical residues in the sequences are underlined in black. Conserved protein functional domain of the deduced *Hi*ADO was identified using the Conserved Domain search feature at NCBI and is depicted in orange (PCO_ADO). GenBank accession numbers are given in Table S1. Figure S2: *H. illucens* cysteine dioxygenase (*Hi*CDO) protein sequence. Multiple sequence alignment of cysteine dioxygenases from Diptera. The conserved residues in the amino acid sequences are highlighted in black; identical residues in the sequences are underlined in black. Conserved protein functional domain of the deduced *Hi*CDO was identified using the Conserved Domain search feature at NCBI and is depicted in blue (CDO_I). GenBank accession numbers are given in Table S1. Figure S3: *H. illucens* cysteine sulfonate decarboxylase (*Hi*CSAD) protein sequence. Multiple sequence alignment of cysteine sulfonate decarboxylases from Diptera. The conserved residues in the amino acid sequences are highlighted in black; identical residues in the sequences are underlined in black. Conserved protein functional domains of the deduced *Hi*CSAD were identified using the Conserved Domain search feature at NCBI and are depicted in blue (DOPA_deC_like) and green (GadA). GenBank accession numbers are given in Table S1. Figure S4: *H. illucens* glutamate decarboxylase (*Hi*GAD) protein sequence. Multiple sequence alignment of glutamate decarboxylases from Diptera. The conserved residues in the amino acid sequences are highlighted in black; identical residues in the sequences are underlined in black. Conserved protein functional domains of the deduced *Hi*GAD were identified using the Conserved Domain search feature at NCBI and are depicted in purple (Pyridoxal_deC) and green (GadA). GenBank accession numbers are given in Table S1. Table S1: List of the species and accession numbers of the protein sequences used in protein alignment and phylogenetic analyses. Table S2: List of primer sequences used in this study.
