# Supplementary material for: Waste Valorization via Hermetia Illucens to Produce Protein-Rich Biomass for Feed: Insight into the Critical Nutrient Taurine
Source: Animals (Basel). 2020 Sep 21;10(9):1710. doi: 10.3390/ani10091710 (PMC7552637; doi:10.3390/ani10091710)
Supplement: Supplementary file 1 [file animals-10-01710-s001.zip › Supplementary -TableS1.docx]

**Table 1.** List of the species and accession numbers of the protein sequences used in protein alignment and phylogenetic analyses.

| **Species** | **GenBank Accession No./Accessions/accession numbers** | | | |
| --- | --- | --- | --- | --- |
|  | **ADO** | **CDO** | **CSAD** | **GAD** |
|  |  |  |  |  |
| *Hermetia illucens* | MT180129 | MT180130 | MT180131 | MT180132 |
| *Bactrocera dorsalis* | XP_011198104.1 | XP_011207266.1 | XP_011199833.1 | XP_011201394.1 |
| *Bactrocera latifrons* | XP_018788081.1 | XP_018783155.1 | XP_018783466.1 | XP_018801723.1 |
| *Bactrocera oleae* | XP_014085146.1 | XP_014092267.1 | XP_014089047.1 |  |
| *Ceratitis capitata* | XP_004523823.1 | XP_012155781.1 | XP_004533948.1 | XP_004525301.1 |
| *Drosophila bipectinata* | XP_017864367.1 | XP_017092655.1 | XP_017108775.1 | XP_017092368.1 |
| *Drosophila erecta* | XP_001971370.1 | XP_026836311.1 | XP_001969575.1 |  |
| *Drosophila hydei* | XP_023174226.2 |  | XP_023163833.2 |  |
| *Drosophila melanogaster* |  |  |  | NP_523914.2 |
| *Drosophila navojoa* | XP_017956873.1 |  | XP_030244339.1 |  |
| *Drosophila novamexicana* | XP_030570068.1 | XP_030558126.1 | XP_030569880.1 |  |
| *Drosophila serrata* | XP_020814695.1 |  | XP_020810126.1 |  |
| *Drosophila willistoni* | XP_002065723.1 | XP_002063538.1 | XP_002066752.1 | XP_015033292.1 |
| *Lucilia cuprina* | XP_023295661.1 | XP_023302836.1 | XP_023301249.1 | XP_023301136.1 |
| *Musca domestica* | XP_005176921.1 | XP_011291169.1 | XP_005188956.2 | XP_011296520.1 |
| *Rhagoletis zephyria* | XP_017464925.1 | XP_017483358.1 | XP_017483023.1 |  |
| *Stomoxys calcitrans* | XP_013114268.1 | XP_013097303.1 | XP_013110104.1 | XP_013117159.1 |
| *Zeugodacus cucurbitae* | XP_011195667.1 | XP_011190605.1 | XP_011190747.1 | XP_011179703.1 |
|  |  |  |  |  |
| *Homo sapiens* | NP_116193.2 | NP_001792.2 | NP_057073.4 | NP_000808.2 |
| *Mus musculus* | NP_001005419.2 | NP_149026.1 | XP_017172101.1 | NP_032103.2 |
| *Danio rerio* | NP_001008634.1 | NP_957035.2 | NP_001007349.1 | NP_919400.1 |
