# Supplementary material for: Waste Valorization via Hermetia Illucens to Produce Protein-Rich Biomass for Feed: Insight into the Critical Nutrient Taurine
Source: Animals (Basel). 2020 Sep 21;10(9):1710. doi: 10.3390/ani10091710 (PMC7552637; doi:10.3390/ani10091710)
Supplement: Supplementary file 1 [file animals-10-01710-s001.zip › Supplementary-Table S2.docx]

**Table 2.** List of primer sequences used in this study.

| **Gene** | **Sequence (5’- 3’) -Forward** | **Sequence (5’- 3’) -Reverse** |  |
| --- | --- | --- | --- |
| **Primers used in cloning *H. illucens* cDNAs** | |  |  |
| ***ado*** | ATGTCTCHVCTATTBGCT | ATCGCRGTANAAGTHCCNC |  |
| ***cdo*** | ATGGCNCWCAATRAAGCYG | CTGTTHYTGTCGHACGCCA |  |
| ***csad*** | GDBCABGAGAAGTTYCTTC | CAAGTHTGCACCAAGDCNCTC |  |
| ***gad*** | ATGTCGMTSAATCCAARG | CAAATCATCHCCYAAYCGATG |  |
|  |  |  |  |
| **Primers used for qPCR** | |  | **E (%)** |
| ***ado*** | ACTATGCCGCTCCACGAT | ACCGCCGTGATTTCGTG | 99.6 |
| ***cdo*** | ATGACCACGCCGACTCC | ATGGTGGACAGTAAAGATGAAG | 100.8 |
| ***csad*** | GTTCAACATTCCTTACACGACA | CGCATTGGATATGTTTGTCAC | 99.9 |
| ***gad*** | CAATCAAATGTCTGCGGAGTA | TAATCGGTCCTGCTGTCGTT | 100.3 |
| ***16s* ^a^** | AGCCTGCCCACTGATTGTTT | CGTTCAGCCATTCATACAAGC | 99.7 |
| ***18s* ^b^** | GGAAGCGTATTATCGGTGGAGT | CAATGCCCCCAACTGCTTCTA | 99.9 |
| ***ef1-α ^c^*** | TATGGTTGTCGTCTTCGCC | GGATGGTTGAGCACGATGA | 99.6 |

E, qPCR Efficiencies; ^a, b, c^, GenBank accessions: KC177455.1, GQ465779.1 and GQ465787.1, respectively.
