# Supplementary figures and images for: Waste Valorization via Hermetia Illucens to Produce Protein-Rich Biomass for Feed: Insight into the Critical Nutrient Taurine
Source: Animals (Basel). 2020 Sep 21;10(9):1710. doi: 10.3390/ani10091710 (PMC7552637; doi:10.3390/ani10091710)

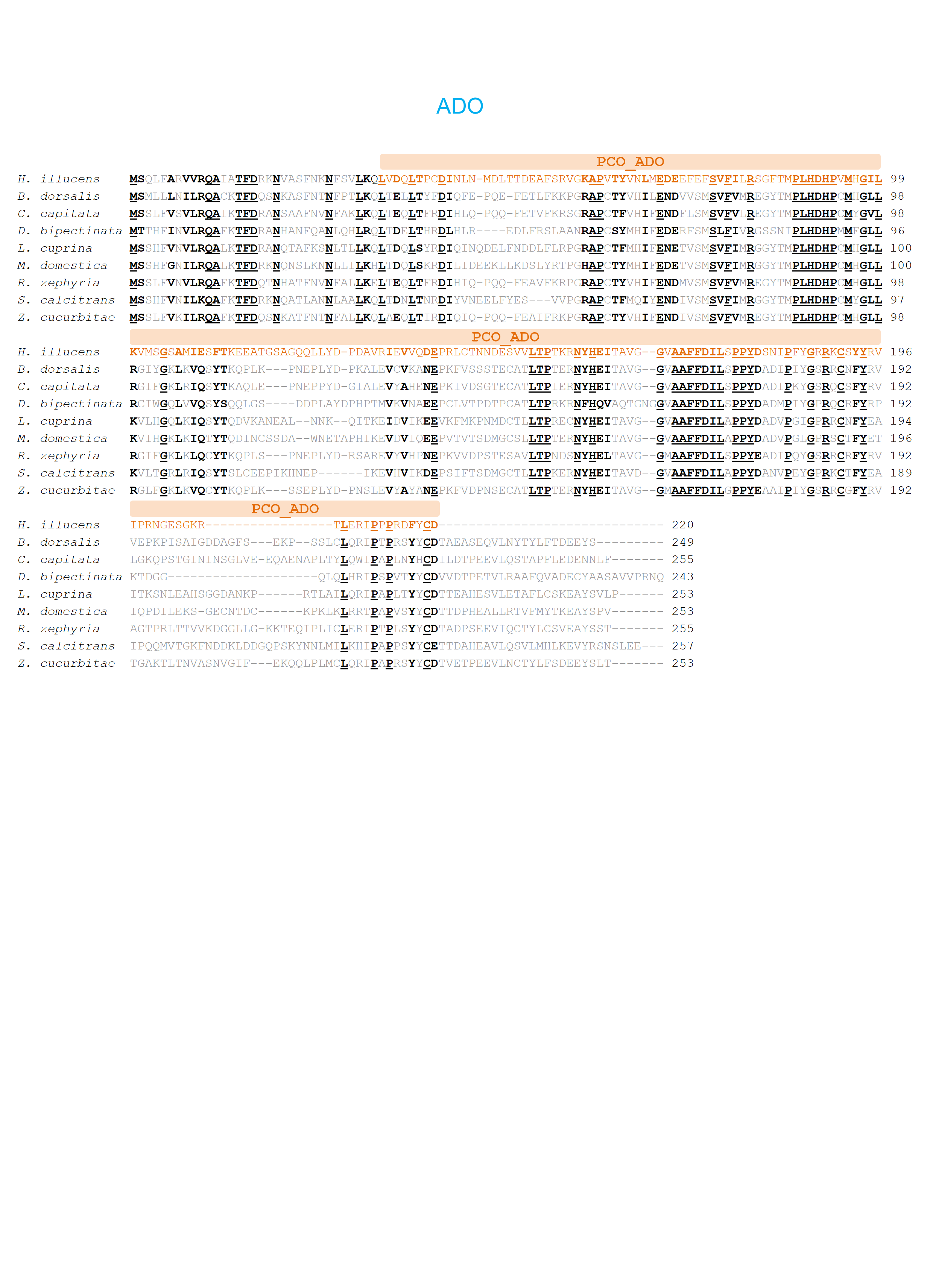

Supplement: Supplementary file 1 [file animals-10-01710-s001.zip › Supplementary material_Fig.S1.tif]

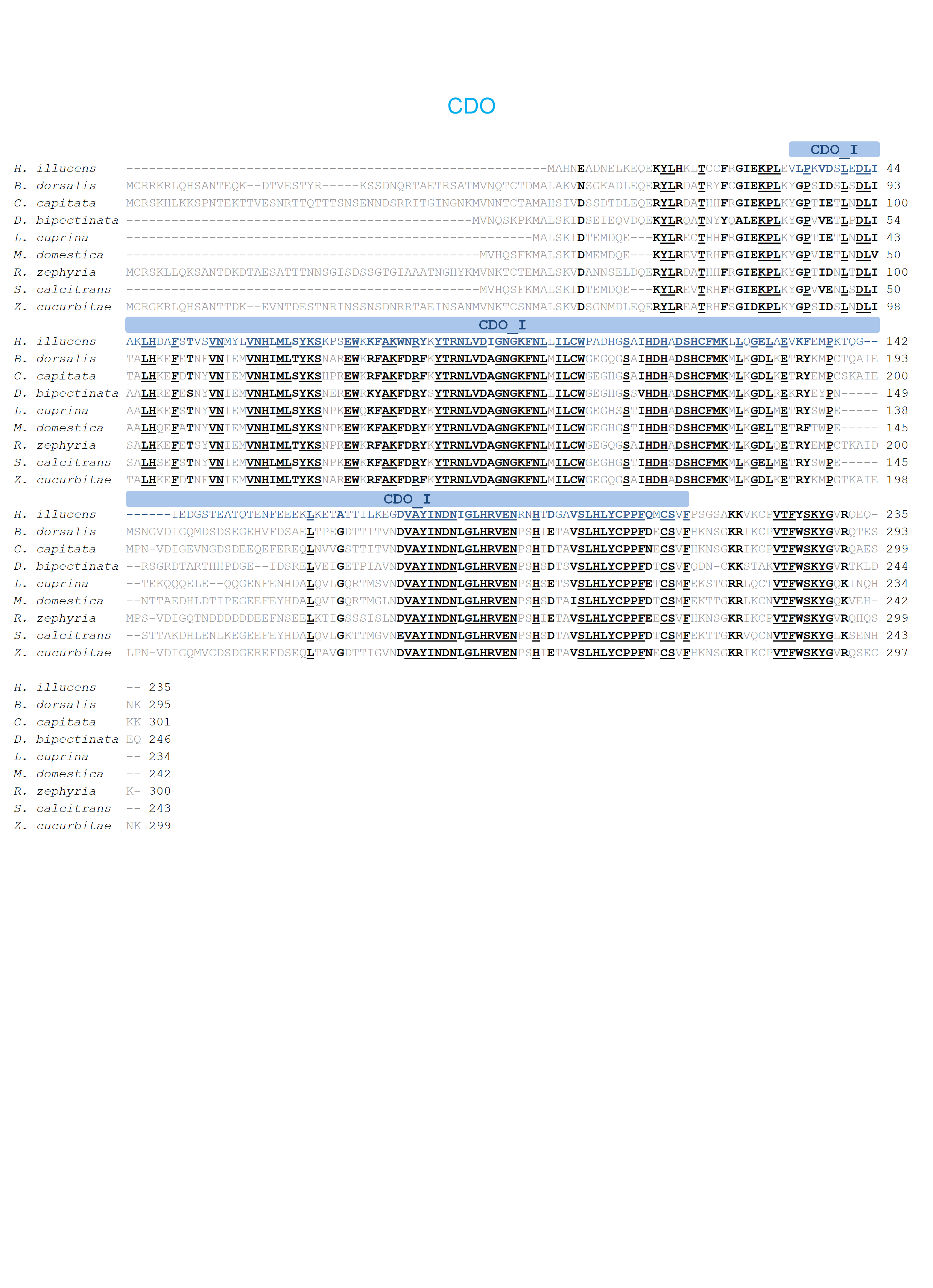

Supplement: Supplementary file 1 [file animals-10-01710-s001.zip › Supplementary material_Fig.S2.tif]

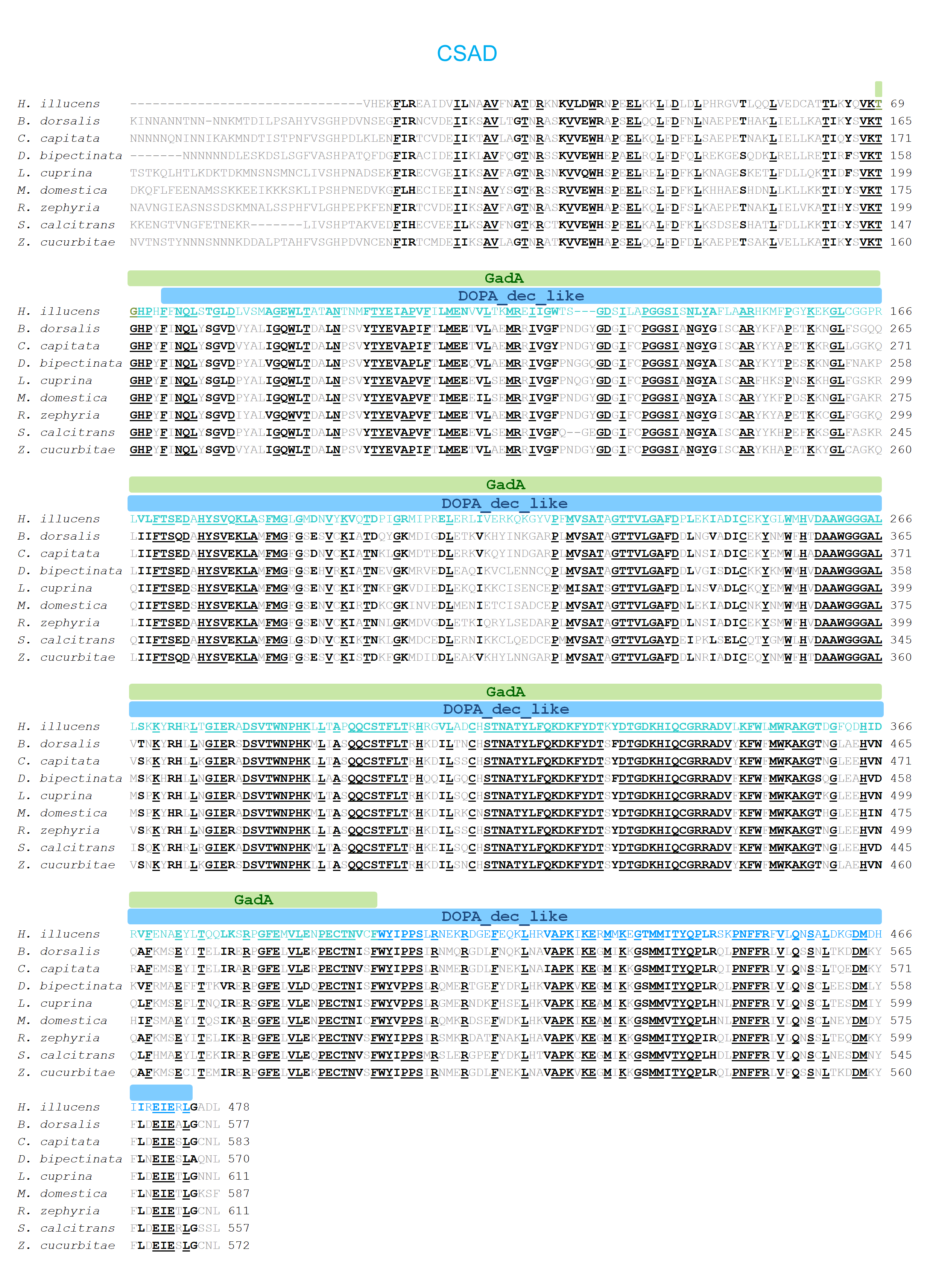

Supplement: Supplementary file 1 [file animals-10-01710-s001.zip › Supplementary material_Fig.S3.tif]

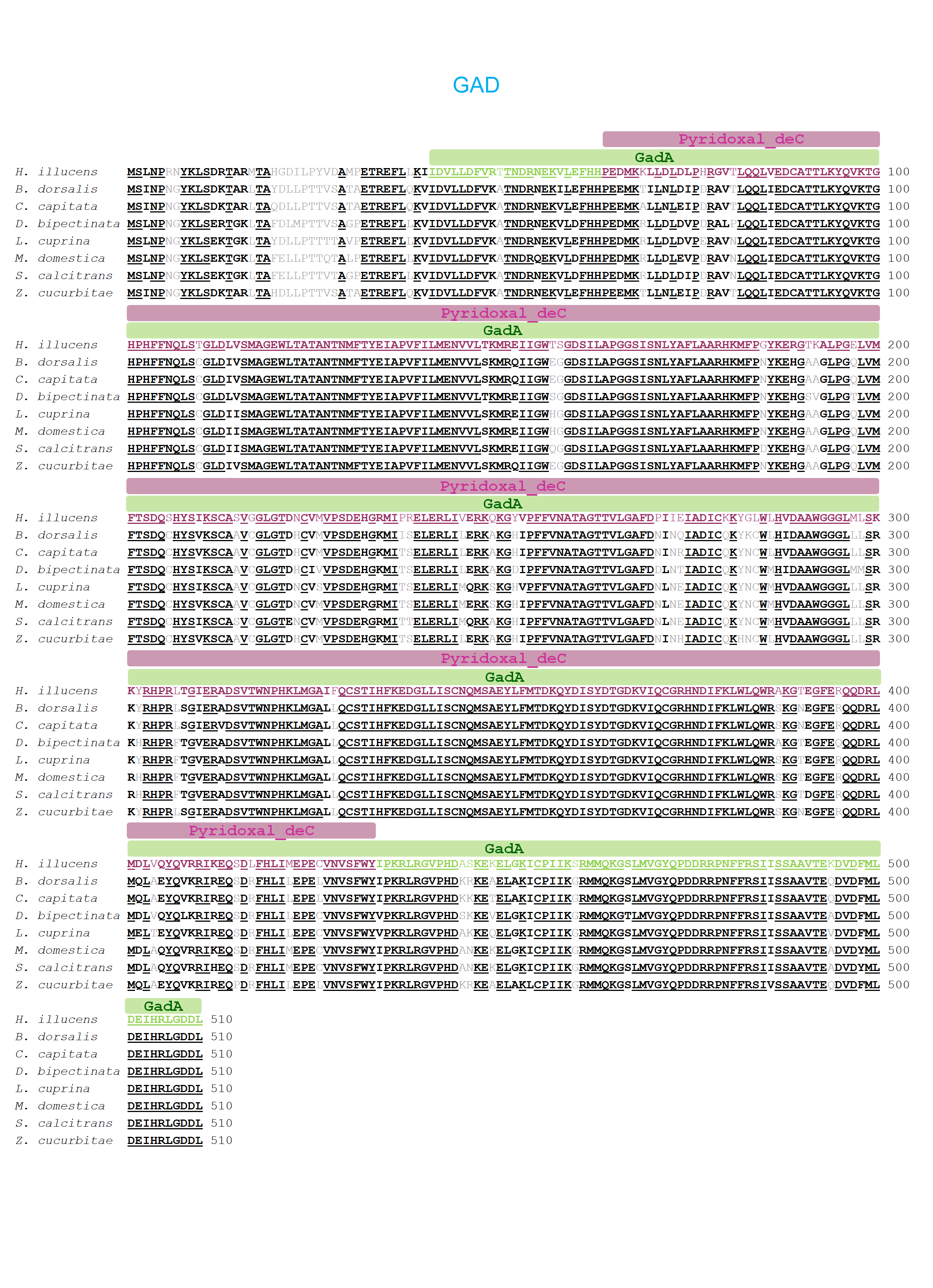

Supplement: Supplementary file 1 [file animals-10-01710-s001.zip › Supplementary material_Fig.S4.tif]
